# The Role of Visfatin (Adipocytokine) Biomarker in Oral Health and Diseases among Nonobese Indian Population: A Proteomic Assay

**DOI:** 10.1055/s-0041-1728690

**Published:** 2021-05-21

**Authors:** Amita Coutinho, Neethu Reddy, Anirban Chatterjee, Mahamad Irfanulla Khan

**Affiliations:** 1 Department of Periodontics, The Oxford Dental College, Bangalore, Karnataka, India; 2 Department of Orthodontics and Dentofacial Orthopedics, The Oxford Dental College, Bangalore, Karnataka, India

**Keywords:** visfatin, biomarker, gingivitis, periodontitis, saliva, diagnostic marker

## Abstract

Visfatin is an adipocytokine and a potential biomarker encoded by the nicotinamide phosphoribosyltransferase gene. It belongs to the nicotinic acid phosphoribosyltransferase family and involved in various metabolic processes and aging. The aim of this study was to evaluate the role of visfatin biomarker in oral diseases like periodontitis. A total of 60 patients (20–50 years) were included in this study, and they were divided into three groups. Group I consisted of 20 subjects with healthy periodontium, group II consisted of 20 subjects with generalized moderate gingivitis, and group III consisted of 20 subjects with generalized periodontitis. The clinical periodontal parameters, including plaque index, gingival index, probing pocket depth, and clinical attachment levels, were recorded, and saliva samples were collected. Salivary visfatin concentrations were assessed using standard enzyme-linked immunosorbent assay. The results of the study showed that the visfatin concentrations were higher in patients with gingivitis and periodontitis compared with those of healthy individuals. Visfatin was found highest in group III (38.22 ± 3.38 ng/mL) followed by group II (26.66 ± 2.24 ng/mL) and the group I (25.60 ± 2.19 ng/mL). Thus, salivary visfatin is a potential inflammatory biomarker and acts as a mediator in the pathogenesis of periodontal disease and, might serve as a diagnostic and therapeutic biomarker in oral diseases like periodontitis.

## Introduction

A biomarker is “a substance that is measured objectively and evaluated as an indicator of normal biologic or pathologic process or pharmacologic responses to a therapeutic intervention.” 1 Biomarkers, whether produced by normal healthy individuals or by individuals affected by any specific systemic diseases, are telltale molecules that could be used to monitor underlying health status, disease onset, and treatment response. Saliva, when considered as a biomarker is an important physiologic fluid, and is rapidly gaining popularity as a diagnostic tool. Saliva contains both host-derived and microbial-derived factors, including several enzymes that degrade proteins, proteoglycans, lipids, and carbohydrates and also enzymes in saliva can originate from cells in salivary glands, microorganisms, epithelial cells, and neutrophils. Saliva, as a mirror of oral and systemic health, is a valuable source for clinically relevant information because it contains biomarkers specific for the unique physiologic aspects of periodontal diseases. 2

Adipose tissue is composed mostly of adipocytes that produce a variety of cytokines and inflammatory molecules, commonly referred to as adipocytokines such as adiponectin, leptin, visfatin, interleukin-6 (IL-6), monocyte chemoattractant protein-1, resistin, tumor necrosis factor-α (TNF-α), and vaspin that regulate different inflammatory processes. These factors influence insulin resistance and are thought to play a role in inflammation, and immune responses and are involved in the pathophysiology of periodontitis. 3

Visfatin is an adipokine, also known as Pre B cell colony enhancing factor, encoded by the nicotinamide phosphoribosyltransferase (NAMPT) gene (located on 7q22.3, OMIM ID: 608764). The structural gene part is composed of 11 exons and 10 introns, which encode 491 amino acids. The protein belongs to the nicotinic acid phosphoribosyltransferase family and is thought to play an important role in immune response and inflammation. 4 5 Visfatin was first reported as a novel adipocytokine secreted preferentially by visceral fat tissue compared with subcutaneous fat in humans and mice, though subsequent reports indicated it is expressed in all adipose tissue and has insulin-mimetic properties. 6

Expression of visfatin is found to be positively regulated in response to microbial stimulation by B cells, T cells, monocytes, macrophages, and neutrophils. 7 8 9 It has been reported that the expression of visfatin is upregulated in a variety of acute and chronic inflammatory diseases such as rheumatoid arthritis, 10 sepsis, 11 acute lung injury, 12 inflammatory bowel disease, 13 diabetes mellitus, 14 aging, 15 metabolic syndrome, and obesity, 16 nonalcoholic fatty liver disease, 17 where there is persistence of inflammation by inhibition of neutrophil apoptosis. Also, it has been shown that visfatin synthesis is regulated by some cytokines such as IL-1β, TNF-α, IL-6, and by lipopolysaccharides. 11 18 In a study, during polyclonal immune responses, visfatin was found to be increased in lymphocytes and stimulated their proliferation. 19 So, visfatin appears to be a key cytokine involved in chronic inflammatory diseases and immune responses.

Several studies reported that the plasma visfatin concentration is significantly increased in type 2 diabetes and/or obese individuals as well as in obese nondiabetic children compared with lean control children influenced by several factors such as age, gender, and body mass index (BMI). 20 21

Advances in oral and periodontal disease diagnostic research are moving toward methods whereby periodontal risk can be identified and quantified by objective measures such as biomarkers. Thus, the aim of this study was to evaluate the role of visfatin biomarker in oral diseases like periodontitis.

## Materials and Methods

### Sample Selection

A total of 60 patients (20–50 years) who visited the Department of Periodontics, The Oxford Dental College, Bangalore, India, were recruited in the study after satisfying the inclusion and exclusion criteria. These subjects were divided into three groups according to the 2017 World Workshop Classification of periodontal diseases and periimplant diseases and conditions. 22 Group I consisted of 20 systemically healthy subjects with healthy periodontium (with no attachment loss and probing depth ≤ 3mm), group II consisted of 20 systemically healthy subjects with generalized moderate gingivitis (with a generalized probing depth of ≤3mm, no attachment loss and generalized bleeding on probing), group III consisted of 20 systemically healthy subjects with generalized periodontitis (clinically and radiographically with generalized moderate periodontitis, with a pocket depth of 5 to 8mm and with moderate alveolar bone loss and clinical attachment loss of 3 to 4 mm).

### Inclusion and Exclusion Criteria

Patients with at least 20 natural teeth with an age group of 25 to 50 years, no systemic disease with BMI of 18.5 to 29.9 kg/m ^2^ were included in the study. Patients with a history of periodontal therapy during the previous 6 months, any systemic disorders that would influence the course of periodontal disease or treatment; patients using glucocorticoids, bisphosphonates, antibiotics, and immunosuppressant medication during the preceding 6 months, menstruating, pregnant, and lactating women; patients undergoing orthodontic therapy; patients with oral mucosal inflammatory conditions; and obese patients with BMI ≥30kg/m ^2^ were excluded from the study.

The study protocol was approved by the Institutional Ethics Committee (No. 433/2015–16) of The Oxford Dental College. All subjects were given a detailed verbal and written description of the study and, written informed consent was obtained from all patients prior to the commencement of the study.

### Clinical Examination

Patients were selected for each group after a brief and precise case history recording that included patient's chief complaint, medical and dental history, clinical examination and radiographic examination. All the clinical measurements gingival index, 23 plaque index, 24 probing pocket depth, 25 and clinical attachment levels 26 were performed by a single operator using sterile mouth mirror and UNC-15 periodontal probe. Orthopantomographs were taken to confirm the bone loss, and BMI 27 was recorded. Clinical parameters were recorded 1 day before saliva sample collection to avoid stimulation of the sample and its contamination with blood.

### Method of Salivary Sample Collection

Unstimulated whole saliva was collected according to Navazesh 28 method on the second day after baseline measurement. According to this method, the patient was advised to avoid food and beverages at least 1 hour before the test session following which the patients were asked to rinse the mouth with water to remove food residues. The patient was then asked to relax for 5 minutes to avoid sample dilution before collection. During sample collection, patients were instructed to minimize movements of the mouth, lean their head forward, and get the Eppendorf tubes close to their mouth that is slightly open, to allow saliva to drain into the tube. Following sample collection, the samples were refrigerated immediately and were transported in an icebox for storage at or below –80°C, for further analysis of visfatin levels using an enzyme-linked immunosorbent assay (ELISA) kit.

### Biomarker Analysis

The concentration of visfatin from saliva was determined with the human visfatin ELISA Kit (Ray Biotech Life, Georgia, USA) according to the manufacturer's instructions using an ELISA. It is based on the principle that the microplate in the kit is precoated with antirabbit secondary antibody. After a blocking step and incubation of the plate with antivisfatin antibody, both biotinylated visfatin peptide and peptide standard or targeted peptide in samples interact competitively with the visfatin antibody. Uncompleted (bound) biotinylated visfatin peptide then interacts with streptavidin-horseradish peroxidase (SAHRP), which catalyzes a color development reaction. The intensity of colorimetric signal is directly proportional to the amount of biotinylated peptide-SAHRP complex and inversely proportional to the amount of visfatin peptide in the standard or samples. This is due to the competitive binding to visfatin antibody between biotinylated visfatin peptide and peptides in standard or samples. A standard curve of known concentration of visfatin peptide can be established, and the concentrations of visfatin peptide in the samples were calculated accordingly. The results of the visfatin assay were expressed as ng/ml for concentrations.

## Statistical Analysis

Statistical analysis was performed using commercially available software (SPSS 22.0, SPSS Inc., Chicago, Illinois, United States). Power analysis was based on the supposition that a mean difference of 0.5 mm in PD should be detected at a significance level of 0.05, and the desired study power of at least 80%. One-way analysis of variance test followed by Tukey's post hoc analysis was used to compare the mean values of various clinical parameters and salivary visfatin levels between three groups. Pearson correlation to assess the relationship of periodontal parameters, BMI, and waist to hip ratio with salivary visfatin levels during pretreatment and post-treatment periods in different study groups. The level of significance was set at *p* < 0.05.

## Results

In this study, we have evaluated salivary visfatin concentrations in periodontally healthy, generalized moderate gingivitis, and periodontitis subjects. All the groups were matched in terms of age ( *p* = 0.005) and gender ( *p* = 0.48). Visfatin was detected in all samples. The levels were highest for periodontitis group (38.22 ± 3.38 ng/mL) followed by the gingivitis group (26.66 ± 2.24 ng/mL) and periodontally healthy subjects (25.60 ± 2.19 ng/mL). Also, the visfatin levels in both normal weight (33.14 ng/mL) and overweight (34.18 ng/mL) subjects were almost equivocal which implies that visfatin levels increase during periodontal inflammation irrespective of the BMI.

## Discussion

The presence of visfatin/NAMPT in white blood cells and tissue-bound macrophage suggests an important role in the regulation of immune and defense functions. 29 Pradeep et al reported increased levels of visfatin in gingival crevicular fluid (GCF) in patients with periodontitis. 30

In the present study, the mean age, for the group I, group II, and group III, was 27.10 ± 3.92, 33.65 ± 4.90, and 35.90 ± 8.89 years, respectively. The influence of age on the visfatin concentration was minimized by selecting the subjects within the specified age group (25–45 years) since age is a known risk factor for both periodontitis and visfatin expression. 31

**Fig. 1:**
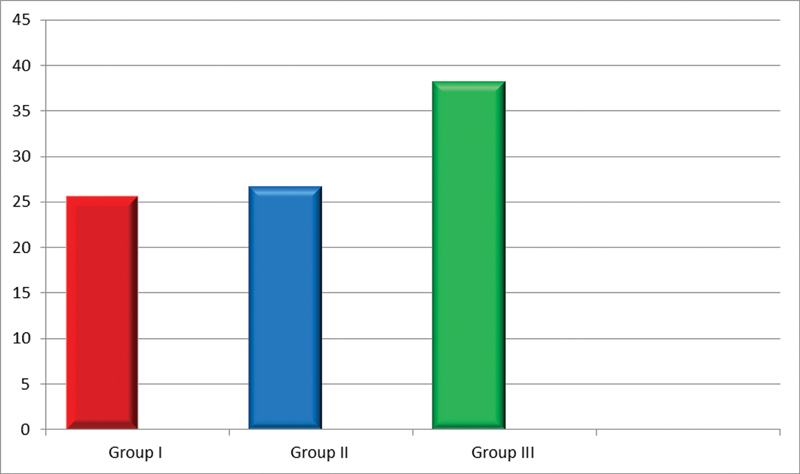
Comparison of salivary visfatin levels in group I, II, and III.

In the present study, the mean baseline salivary visfatin levels in group II and group III were 26.66 ± 2.24 and 38.22 ± 3.38 ng/mL ( Table 1 , Fig. 1 ). Similar observations were made by Pradeep et al, 30 where visfatin levels in serum and GCF increased proportionately with the severity of the disease. These changes were seen since visfatin is actively secreted by predominant cells involved in periodontal disease activity and variability in visfatin concentrations within patients of each group could be attributed to the role of visfatin in different stages of a disease process. Also, Tabari et al 32 speculated that changes in the microbial composition and the ongoing inflammatory process in the pocket environment have a close relationship with the visfatin levels in saliva.

**Table 1:** Comparison of salivary visfatin levels in group I, II, and III

Group I		Group II		Group III		*p* -Value	Intergroup comparison
Mean	SD	Mean	SD	Mean	SD		
25.60	2.19	26.66	2.24	38.22	3.38	<0.001 a	Group I vs. III ( *p* < 0.001**) Group II vs. I ( *p* = 0.59) Group III vs. II ( *p* < 0.001**)

Abbreviation: SD, standard deviation.

a *p* < 0.001 highly significant.

The mean levels of salivary visfatin in periodontitis subjects were 38.32 ± 3.83 ng/mL that are in accordance with the study done by Nokhbehsaim et al, 33 who demonstrated that visfatin stimulates the production of C–C motif chemokine ligand 2 and matrix metalloproteinase-1 in the periodontal ligament cells and thus can lead to inflammation of periodontium and destruction of connective tissue. This production of visfatin in the periodontal ligament cells could be induced by periodontal pathogens, *Porphyromonas* *gingivalis* and *Fusobacterium* *nucleatum* , and proinflammatory cytokine, IL-1β. Hence, the microbial and inflammatory signals can use visfatin for its destructive effects on the periodontium. In addition, visfatin can result in the production of proinflammatory and matrix-destructing cytokines, and therefore, interfere with the regenerative capacity of periodontal ligament cells. 34

**Fig. 2:**
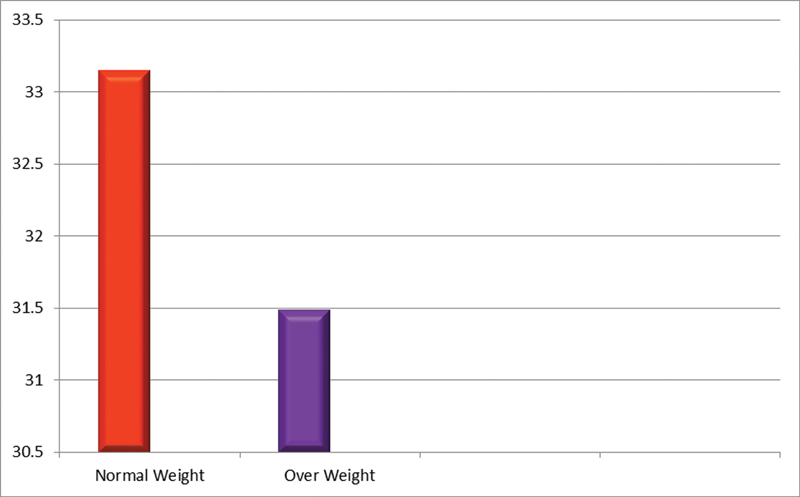
Mean visfatin levels in normal and overweight subjects in all three groups.

In the previous studies, the correlation between obesity and visfatin levels is confirmed, 33 showing that visfatin levels were higher in obese individuals as compared with normal-weight controls. In this study, we tried to find the correlation between salivary visfatin levels and normal and overweight patients (based on BMI) that was not compared in the previous studies to date. The results we got were surprising. Visfatin levels in the normal weight patients were 3.31482 ng/mL, and the visfatin levels in the overweight patients were 3.1487ng/mL. This shows that at baseline, visfatin levels increase irrespective of whether the patient is normal weight or overweight. These values were statistically significant ( *p* < 0.001) ( Table 2 , Fig. 2 ). This negative correlation between BMI and salivary visfatin levels may be because of (i) ethnic heterogeneity may affect visfatin level or visfatin sensitivity and (ii) genetic association analysis found that single-nucleotide polymorphism locus and two other loci located at the intron region of the visfatin gene were associated with lipid metabolism, which indicates that this gene may account for some variation in the concentrations of visfatin, which is in accordance with the study done by Jian et al. 35

**Table 2:** Relationship between BMI (kg/m ^2^ ) and salivary visfatin levels

BMI category	Mean	Sample ( *n* )	SD	SE mean	Significance
Normal weight	33.148	33	6.7098772	1.3991061	*p* < 0.001
Overweight	31.487	27	6.2788810	1.5228523	

Abbreviations: BMI, body mass index; SD, standard deviation; SE, standard error.

This study has a few limitations. First, the study sample size could have been larger and second, the study could have included obese patients and could have been done for a longer duration. Therefore, further multicenter longitudinal studies have to be conducted to critically evaluate the results of the current study, with a larger sample population.

Despite these limitations, our study confirmed that salivary levels of visfatin were increased in the order of disease severity; least in periodontally healthy, followed by gingivitis and most in periodontitis subjects. Hence, visfatin can be used as a diagnostic marker in periodontal disease.

## Conclusion

The results of this study suggest that salivary visfatin concentrations were higher in patients with gingivitis and periodontitis compared with periodontally healthy individuals. Therefore, salivary visfatin is a potential inflammatory biomarker and acts as a mediator in the pathogenesis of periodontal disease and may be considered as a diagnostic biomarker of oral diseases like periodontitis.
